# Evaluation of the Antidiabetic Potential of Extracts of *Urtica dioica*, *Apium graveolens*, and *Zingiber officinale* in Mice, Zebrafish, and Pancreatic β-Cell

**DOI:** 10.3390/plants10071438

**Published:** 2021-07-14

**Authors:** Rosa Martha Pérez Gutiérrez, Alethia Muñiz-Ramirez, Abraham Heriberto Garcia-Campoy, José María Mota Flores

**Affiliations:** 1 Laboratorio de Investigación de Productos Naturales, Escuela Superior de Ingeniería Química e Industrias Extractivas, Instituto Politécnico Nacional, Av. Instituto Politécnico Nacional S/N, Unidad Profesional Adolfo López Mateos, Ciudad de México CP 07708, Mexico; abrahamhgc27@hotmail.com (A.H.G.-C.); josemariamota@yahoo.com (J.M.M.F.); 2 CONACYT/IPICYT-CIIDZA, Camino a la Presa de San José 2055, Col. Lomas 4 Sección, San Luis Potosí CP 78216, Mexico; alethia.muniz@ipicyt.edu.mx

**Keywords:** *Urtica dioica*, *Apium graveolens*, *Zingiber officinale*, diabetes, mice, zebrafish, pancreatic β- RINm5F cell

## Abstract

Medicinal plants are commonly used in the treatment of diabetes, particularly as they contain flavonoids and phenolic compounds. The present study aims to investigate the activities of a polyherbal formulation made from *Urtica dioica*, *Apium graveolens*, and *Zingiber officinale* (UAZ) against streptozotocin–nicotinamide ((STZ-NA)-induced type 2 diabetes in CD1 mice, glucose-induced type 2 diabetes (T2DM) in zebrafish, and high glucose-induced damage in RINm5F pancreatic β-cells. In fasting mice, plasma glucose, glycosylated hemoglobin (HbA1C), lipid hydroperoxides (LOOH), thiobarbituric acid reactive substances (TBARS), and lipid profiles were significantly increased, whereas insulin, enzymatic antioxidants, and carbohydrate metabolic enzymes were altered significantly in diabetic mice. Zebrafish had similar glucose levels, liver enzymes, and lipid profiles compared to mice. The study investigated the effects of the extract in enhancing cell viability, insulin secretion, and reducing lipid peroxidation and intracellular reactive oxygen species (ROS) levels in RINm5F cells damaged by high glucose. All the above biochemical parameters were enhanced in both mice and zebrafish treated; the combined extract UAZ normalized all the biochemical parameters. The medicinal plant extracts, used either separately or in combination, ameliorated the adverse effect of glucose on cell viability and functionality of beta-RINm5F cells.

## 1. Introduction

Diabetes mellitus is a chronic carbohydrate metabolism disease characterized by hyperglycemia, and it is caused by total lack of insulin, insufficient secretion, or synthesis of insulin or peripheral resistance to insulin effect. The prevalence rate of diabetes is increasing exponentially, and the World Health Organization predicts that by the year 2030, diabetes is expected to be the seventh leading cause of death worldwide. This has been attributed to the modern lifestyle, which produces an imbalance between energy intake and energy expenditure [1]. Major advances have been made in anti-diabetic drugs, such as oral hypoglycemic agents and insulin; however, anti-diabetic drugs have adverse effects such as cramps, gastrointestinal irritation, diarrhea, nausea, and flatulence [2]. Thus, it is important to find other options in edible and medicinal plants in order to treat diabetes.

Zebrafish (*Danio rerio*) facilitate studies on the mechanisms of pharmacology at the molecular level thanks to the availability of a complete genome sequence. In addition, several commercially enzyme-linked immunosorbent assay (ELISA) kits have been developed to offer new insights into T2DM in zebrafish; these have been used in the study of various diseases including non-alcoholic steatohepatitis, diabetes complications, atherosclerosis, cancer, and visceral adiposity, etc. [3].

Medicinal plants contain pharmacodynamic bioactive compounds whose additive and synergistic therapeutic effects are beneficial for the management of metabolic disorders. Most pharmaceutical drugs are developed from medicinal plants using local communities’ knowledge and subsequent isolation of the main active ingredients. However, there is a scarcity of scientifically accurate reviews and experimental evidence regarding the efficacy and safety of medicinal plants. The systematic study of medicinal plants and the investigation of their biologically active phytochemical compounds for the management of metabolic disorders have emerged as a key development in modern medicine [4]. Unlike current medicinal systems, polyherbal formulations have received more attention due to their multi-targeting capacity with fewer side effects [5]. These drug systems consist of polyherbal preparations that can suppress symptoms associated with various ailments such as obesity, arthritis, cancer, and diabetes, among others.

Several medicinal plants have been studied to prevent diabetes and related diseases. These include *Urtica dioica*, which decreases the level of glucose and fructosamine in alloxan-induced NOD mice [6]; *Zingiber officinale* (ginger), which ameliorates streptozotocin-induced diabetic liver injury in rats [7], controls blood sugar in patients with type 2 diabetes mellitus [8], and *Apium graveolens* (celery) which enhance blood glucose and insulin levels in older people with pre-diabetes [9]. The synergistic effects of polyherbal formulation consisting of *U. dioica*, *A. graveolens*, and *Z officinale* on diabetes have not been previously studied. Thus, this study aims to evaluate whether this polyherbal formulation has synergistic effects and can protect the metabolism of the STZ-induced diabetic mice, glucose-induced diabetic zebrafish, and pancreatic β-cells from high glucose-induced damage.

## 2. Materials and Methods

### 2.1. Chemicals

All chemicals for the present study were purchased from Sigma Aldrich (St. Louis, MO, USA).

### 2.2. Plant Collection

*U. dioica*, *A. graveolens*, and *Z. officinale* plants were purchased from Central de Abastos, CDMX, (México). The plant material was authenticated in the Escuela Nacional de Ciencias Biológicas-IPN, Mexico. The plant material was deposited at the Department of Botany (voucher numbers CB12478, CB12476, and CB12479, respectively). The dried plants were ground to a particle size that passed through a 0.5 mm sieve using an electric grinder. The combined products (UAZ) were prepared by mixing the three plants at a weight ratio of 1:1:1 (*w*/*w*).

### 2.3. Preparation of Extracts

Each of the fresh plants was shadow-dried and grinded into powder to produce the polyherbal formulation (1000 g). The plants were extracted with hydroalcoholic solution, 50% ethanol, and 50% water for 1 h in a Soxhlet apparatus to extract mainly flavonoids and phenolic acids. The plant extracts were then dried to 50 °C in a vacuum rotary evaporator and the residue was studied for its pharmacological activity on animals.

### 2.4. Identification of Phytochemicals

The extracts of the three plants (*U. dioica*, *A. graveolens*, and *Z. officinale*) were analyzed by LC–MS/MS (UHPLC, Agilent Technologies series 1290, Santa Clara, CA, USA). Chromatographic separation was performed at 38 °C on a C18 analytical column (2.1 mm × 100 mm, 3.5 μm) and Waters Symmetry (Waters Technologies Corporation, Milford, MA, USA). A gradient of acetonitrile and water (A) with or without formic acid (B, 0.1%) was applied from 10−18% B (0–4 min); 18−20% B (4−9 min); and 20−20% B (9−10 min). MS spectra were conducted on an Agilent 6460 triple quadrupole mass spectrometer equipped with electrospray ionization (ESI) source, in positive ionization mode (Agilent Corporation, Santa Clara, CA, USA). The following were used for the MS spectra: drying gas temperature of 325 °C, drying gas flow of 10 L/min^−1^, sheath gas temperature of 250 °C, sheath gas flow of 10 L·min^−1^, nebulizer pressure of 40 psi, and a capillary voltage of 4.0 kV in a mass range of 100–2500 *m*/*z*. Data collection and processing were conducted using a Mass Hunter Workstation 05.00 (Agilent Technologies, Santa Clara, CA, USA).

### 2.5. Determination of Flavonoid Content

To estimate the amount of flavonoid, 1 mL of the plant extract was added to 4 milliliters of distilled water and stirred with 0.3 mL sodium nitrite solution (5%) and 0.3 mL aluminum chloride solution (10%). After the tubes were incubated for 5 min at ambient temperature, 2 mL of sodium hydroxide (1 M) was added. The tubes were filled up to 10 mL using distilled water, then the absorbance value was measured at 510 nm.

The total flavonoid contents were determined as rutin equivalent per 100 g dry weight (g RE.100 g−1 DW) using a calibration plot with rutin.

### 2.6. Experimental Mice

CD1 mice (males) of six weeks old were used in this study. Their body weight was 24–29 g at the beginning of the experiments. They were acclimated under a 12 h light/dark cycle (lights on at 08:00 a.m.) in separate cages. They were given water ad libitum and normal chow (Purina) for a week before the experiment. The animals were kept at a humidity of 45–55% and a constant temperature of 20–22 °C in separate cages with metallic walls. The front wall of the cages was equipped with a drinking burette. The animals were given water ad libitum and standard pellets as a basal diet, which was freely administered to them.

#### 2.6.1. Induction of Diabetes in Mice

Diabetes was induced in mice fasted overnight by a single intraperitoneal (ip) injection of streptozotocin (STZ; Sigma, St. Louis, MO, USA) in a dose of 45 mg/kg. The STZ was dissolved in citrate buffer as the vehicle (0.1 M, pH 4.5) before use for 15 min, then nicotinamide (110 mg/kg BW in normal physiological saline) was administered [10]. Finally, a solution of 5% glucose was provided to prevent hypoglycemia. Fasting blood glucose levels were measured for 72 h and then the STZ injection was administered with a glucometer in the blood drawn from the tail vein. Diabetes was confirmed in the mice with fasting blood glucose above 250 mg/dl (13.8 mM), which was measured by Accu-Chek Glucometer (Accu-Chek^®^ Active, Roche Diagnostics GmbH, Hannheim, Germany) and expressed in terms of mg/dl.

#### 2.6.2. Experimental Design Mice

All mice were divided into seven groups with eight mice in each group:

Group I: normal control (given water and standard diet for 4 weeks); Group II: diabetic mice control (ZTZ); Group III: diabetic mice treated with *U. dioica* extract (U; 250 mg/kg); Group IV: diabetic mice treated with *A. graveolens* extract (A; 250 mg/kg); Group V: diabetic mice treated with *Z. officinale* extract (Z; 250 mg/kg); Group VI: diabetic mice treated with polyherbal formulation (UZA; 250 mg/kg); Group VII: diabetic mice treated with Metformin standard drug (Mtf; 100 mg/Kg).

#### 2.6.3. Serum Biochemical Analysis

In all groups, blood samples were collected and centrifuged at 2000× *g* for 20 min to separate the serum for biochemical test. The levels of total cholesterol, high-density lipoprotein (HDL), and triglycerides in the mice serum were evaluated using the kit assay (total cholesterol and triglycerides Home Test Meter Kit Monitor, Solana Health Inc, Del Mar, CA, USA). The activities of fructose-1,6-bisphosphatase hexokinase, glucose-6-phosphatase, and phosphofructokinase in the liver were determined using kit assays from BioVision, (Milpitas, MA, USA). Glycated hemoglobin (HbA1c) was measured by ELISA kit assay (Elabscience, Houston, TX, USA). Superoxide dismutase (SOD), catalase (CAT), and reduced glutathione (GSH), as well as oxidative stress biomarkers, namely thiobarbituric reactive species (TBARS), alkaline phosphatase (ALP), alanine aminotransferase (ALT), and aspartate aminotransferase (AST) were estimated using Elisa diagnostic kits (Abcam, Cambridge, CA, USA). Plasma insulin was assayed by ELISA kit (Boeheringer–Manneheim Kit, Manneheim, Germany).

Lipid hydroperoxide (LOOH) was evaluated using the Jiang et al. [11] method. In brief, 0.1 mL plasma was added to 0.9 mL of Fox reagent (0.8 mg of ammonium iron sulfate, 88 mg of butylated hydroxytoluene, and 7.6 mg of xylenol orange). The mixture was added to 10 mL of sulphuric acid (250 mM) and 90 mL methanol; it was incubated at 37 °C for 30 min and read at 560 nm.

Insulin resistance (HOMA-IR) was calculated with fasting blood glucose and fasting serum insulin at the end of the experimental period using the Wilson and Islam [12] formulas.

HOMA − IR = Insulin U/L × blood glucose (nmol/L)/22.5

### 2.7. Cell Culture

RINm5F cell line (rat insulinoma) was obtained from the American Type Culture Collection (ATCC), USA. The cells were grown in an RPMI-1640 medium supplemented with 50 μmol/L, β- mercaptoethanol, 1 mmol/L sodium pyruvate, 10 mmol/L HEPES, 10% fetal bovine serum (FBS), 2 mmol/L L-glutamine, 100 μg/mL streptomycin, and 100 U/mL penicillin at 37 °C in a humidified atmosphere of 5% CO_2_ and 95% air.

#### 2.7.1. Measure of Cell Viability

RINm5F cells (rat insulinoma cell lines) were seeded (5 × 104 cells/well) and incubated overnight. These were pretreated with various concentrations of the extracts (0–1000 μg/mL) for 24 h. The medium was then removed, and the cells were washed with phosphate buffer solution (PBS). 200 μL of 0.2% crystal violet solution was added to each well and incubated for 10 min at room temperature to be subsequently washed with water; 100 μL 1% SDS was then added to solubilize the stain solution until its color became uniformed. There was dense coloration at the bottom of the wells. The absorbance of the samples was measured at 590 nm in a microplate reader (Spectra MAX, Gemini EM, Molecular Device).

#### 2.7.2. Insulin Secretion Stimulated by Glucose in RINm5F Cells

To evaluate the effect of glucose, RINm5F cells were grown for 48 h and then incubated under basal conditions, UAZ extract (10, 25, 50 µg/mL), or in the presence of high glucose concentrations for 2 h. 25 mM glucose was added in the last 15 min, while the untreated cells were used as control. The cell was rapidly removed and rinsed twice with phosphate-buffered solution. Insulin levels were determined using an insulin enzyme-linked immunosorbent assay kit (EMD Millipore, Billerica, MA, USA).

#### 2.7.3. Lipid Peroxidation (LPO) in RINm5F Cells

Lipid peroxidation was measured by TBARS production. RINm5F Cells (2 × 104 cells/well) were preincubated with glucose (25 mM) for 48 h. These were subsequently incubated with or without UAZ extract (10, 25, 50 µg/mL) for 24 h. 200 μL of each medium supernatant was added to 400 μL of TBARS solution then boiled at 95 °C for 20 min. TBARS concentrations were measured at 532 nm and extrapolated in a serial dilution standard curve of 1,1,3,3-tetra ethoxy propane. TBARS data were expressed as equivalent moles of malondialdehyde (MDA).

#### 2.7.4. Measurement of Intracellular Reactive Oxygen Species (ROS) Level in RINm5F Cells

Intracellular ROS levels were determined by dichlorofluorescein assay [13]. 2,7 -Dichloro dihydrofluorescein fluorescence (2′,7′-DCF-DA) was used to measure the production of ROS. Cells (2 × 104 cells/well) were preincubated with glucose (25 mM) for 48 h, and later incubated with or without UAZ extract (10, 25, 50 µg/mL) for 24 h. The cells were washed with PBS and incubated with 5 μM 2,7-DCF-DA for 30 min at room temperature. The fluorescence intensity was measured in the microplate reader (Spectra MAX, Gemini EM, Molecular Device) at a wavelength of 485 nm (excitation) and 538 nm (emission). The level of intracellular ROS was shown as a percentage of non-treated control.

### 2.8. Experimental Zebrafish

Adult zebrafish (*Danio rerio*) were maintained according to standard protocols. The fish were maintained at a temperature of 28.5 °C using a light schedule of 10 h to 14 h. Fish were fed twice daily using both brine and dry shrimp. All animal experiments were performed according to the protocol approved by the Animal Experiment Committee of Escuela Nacional de Ciencias Biologicas (IPN; Protocol No. 2732).

#### 2.8.1. Induction of Diabetes in Zebrafish

Adult male fish were immersed in 4% glucose (Sigma, St Louis, MI, USA) dissolved in E3 medium for four weeks, while only non-diabetic fish were maintained in E3 medium. As the fish treated with 4% glucose had an optimal survival rate, induced hyperglycemia, and other complications, all the following experiments were carried out under these conditions. In order to evaluate the effects of each plant (U, A, and Z used singly) and the polyherbal formulation (UAZ), glucose fish were treated with all extracts (25 mg/L) for 4 weeks [14]. Metformin was dissolved in fish water to a final concentration of 25 mM. The metformin standard solution was freshly prepared and changed daily. Blood samples were collected after 7 days of exposure to the extracts and metformin. At the end of the treatment, fish were euthanized using ice water.

#### 2.8.2. Experimental Design in Zebrafish

All fish were divided into seven groups consisting of twenty fish in each group. The extracts were dissolved in fish water. The fish were exposed to each extract for 3 h daily over 4 weeks. The fish were grouped as follows: Group I: Normal group; Group II: diabetic control fish (ZTZ); Group III: diabetic fish treated with *U. dioica* extract (U; 25 mg/L); Group IV: diabetic fish treated with *A. graveolens* extract (A; 25 mg/L); Group V: diabetic fish treated with *Z. officinale* extract (Z; 25 mg/L); Group VI: diabetic fish treated with polyherbal formulation (UAZ; 25 mg/L); Group VII: diabetic treated with Metformin standard drug (Mtf; 25 mM).

#### 2.8.3. Effect of UAZ on Liver Enzymes and Thiobarbituric Reactive Species (TBARS) of Zebrafish

The fish livers were homogenized with a phosphate-buffered solution (0.1 M; PBS, pH 7.4) in an ice-cold medium containing 1 mM of dithiothreitol (DTT), 1 mM of ethylenediamine tetra-acetic acid (EDTA), 0.15 M of KCl, and 0.5 M of sucrose. The homogenates were centrifuged at 12,000× *g* and 4 °C for 30 min. In the supernatants, lipid peroxidation (LPO) was determined using the thiobarbituric reactive species (TBARS) test. Thiobarbituric acid can react with end products to produce a pinkish red chromogen, which determines the malondialdehyde that reacts with thiobarbituric acid [15]. Alkaline phosphatase (ALP), alanine aminotransferase (ALT), and aspartate aminotransferase (AST) were estimated using kit methods (Abcam, MA, USA).

#### 2.8.4. Effect of UAZ on Levels of Glucose, Total Cholesterol, and Triglycerides in Serum in Zebrafish

The levels of glucose, total cholesterol, and triglycerides in the serum of the fish were evaluated using the kit assay (total cholesterol and triglycerides Home Test Meter Kit Monitor, Solana Health Inc., CA, USA and Abcam, MA, USA, respectively).

### 2.9. Statistical Analysis

All results were expressed as mean ± standard deviation (SD); the significance between the groups was determined using one-way analysis of variance (ANOVA), followed by Tukey’s multiple comparisons. GraphPad Prism version 7 was used to evaluate the results.

## 3. Results

### 3.1. Phytochemicals

**Figure 1:**
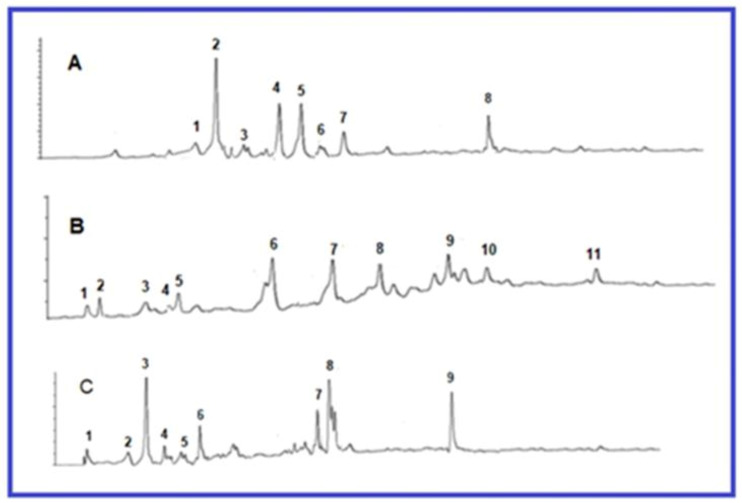
HPLC chromatogram of (**A**) *U. dioica*: isorhamnetin-3-*O*-rutinoside (1); quercetin-3-o-glucoside (2); kaempferol-3-*O*-glucoside or astragalin (3); myrecetin (4); naringin (5); rutin (6); ferulic acid (7); ellagic acid (8); (**B**) *Z. officinale:* morin (1); gallic acid (2); fisetin (3); vanillic acid (4); epicatechin (5); *trans*-cinnamic acid (6); kaempferol (7); naringenin (8); rutin (9); ferulic acid (10); quercetin (11); (**C**) *A. graveolens* chlorogenic acid (1); vanillic acid (2); caffeic acid (3); syringic acid (4); luteolin (5); *p*-coumaric acid (6); rutin (7); ferulic acid (8); quercetin (9).

LC–MS/MS analysis was used to identify the flavonoids and phenolic acid of the studied plants. The results are summarized in Figure 1. Furthermore, they were identified by using UV/vis spectrum and direct comparison reference compounds. In investigating the phytochemical composition of *U. dioica* leaves, it was revealed that the leaves contain phenolic compounds such as ellagic acid, ferulic acid, kaempferol-3-*O*-glucoside (astragalin), myricetin, naringin, quercetin-3-*O*-glucoside, isorhamnetin-3-*O*-rutinoside (9), and rutin. The *Z. officinale* shows the presence of naringenin, rutin, gallic acid, ferulic acid, *trans*-cinnamic acid, fisetin, kaempferol, morin quercetin, and vanillic acid. In addition, *A. graveolens* contains ferulic acid, gallic acid, quercetin, rutin, vanillic acid, caffeic acid, chlorogenic acid, *p*-coumaric acid, luteolin, and syringic acid, which were identified as the main active compounds (Table 1).

### 3.2. Plasma Glucose, Insulin, and Haemoglobin (HbA1c) in Mice

**Figure 2:**
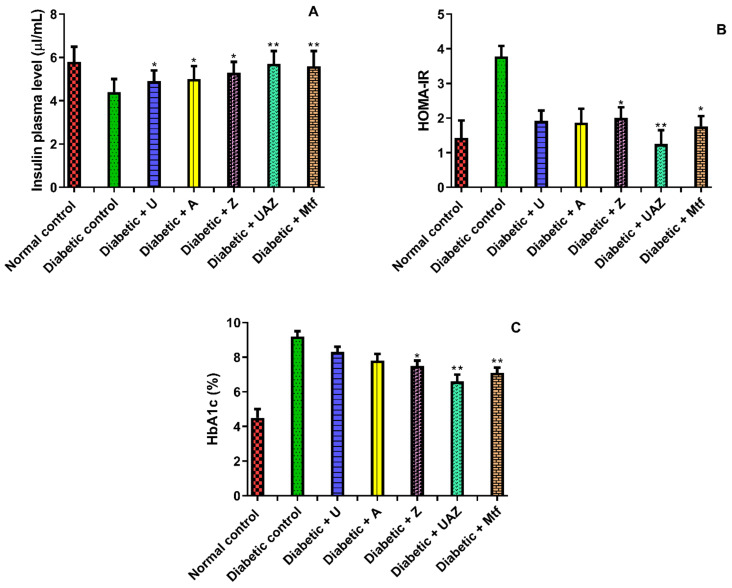
Values are shown as mean ± SD (n = 8). (**A**) Insulin plasma; (**B**) Insulin resistance (HOMA-IR); (**C**) HbA1c over 4 weeks of treatment (250 mg/kg). The animals were fasted 12 h before blood sampling. Metformin (Mtf; 100 mg) was administered as a positive control, and the vehicle was administered as negative control. There is a significant difference compared with the control group * *p* < 0.01 and compared vs. diabetic control group ** *p* < 0.05.

The levels of plasma glucose and insulin of normal and treated mice are shown in Table 2 and Figure 2A, respectively. In diabetic mice, there was a significant (*p* < 0.05) increase in plasma glucose, while the levels of insulin in plasma markedly (*p* < 0.05) decreased in the diabetic control animals compared to the normal group. In diabetic animals treated with UZA or U, A, and Z, there was a significant (*p* < 0.05) increase in insulin (1.3, 1,1, 1.1, 1.2 fold, respectively), while a decrease in plasma glucose levels (68.1%, 53.8%, 55.6% and 58.4%, respectively) was observed. Findings indicate that the polyherbal formulation made plasma insulin and glucose approximate to regular values, while normal mice did not display any significant disturbance in plasma glucose and insulin levels in the experimental period. The insulin resistance (HOMA-IR) result is shown in Figure 2B. The HOMA-IR value has a similarly changing trend with levels of insulin and glucose. The effects of the extracts on HbA1c level in the STZ-induced diabetic mice are shown in Figure 2C, where a significant increase in HbA1c level was observed compared to the control animals. UAZ or U, A, and Z treatments given to STZ animals significantly inhibited (*p* < 0.05) the HbA1c level with values of 28.2%, 9.8%, 15.2%, and 18.5%, respectively. The extracts treatment did not cause a significant change in the HbA1c levels compared to the control diabetic mice.

### 3.3. Effects of U, A, and Z Extracts and Their Combination (UZA) on Carbohydrate Metabolic Enzymes in Mice

The activities of U, A, and Z extracts separately and in combination (UAZ) on carbohydrate metabolic enzymes in the liver of STZ-induced diabetic mice, normal mice, and those treated with extracts are shown in Table 3. The enzymes of glucose metabolism in the liver of the animals such as Glucose-6-phosphatase dehydrogenase (G6PD), Glucose-6-phosphatase (G6P), hexokinase, Phosphofructokinase and Fructose-1, 6-bisphosphatase (F1) were significantly altered in the experimental diabetic mice. The activities of glucose-6-phosphatase dehydrogenase (G6PD) and hexokinase were significantly reduced (*p* < 0.05) in the liver of the diabetic mice. These enzyme activities were restored in the mice treated with UAZ extract and metformin (250 and 100 mg/kg bw, respectively) to that of the normal group. It is demonstrated that there is a significant decrease in both G6P and F1 activities in the hepatic tissues of diabetic mice after being given the extracts orally. It shows that the extracts tend to regulate the carbohydrate metabolic enzymes, thereby reducing hyperglycemic status (Table 3). Phosphofructokinase was significantly reduced in diabetic mice compared to the normal group, while U, A, Z, and UAZ extracts and metformin-treated mice had increased levels (1.26, 1.3, 1.34, 1.53 and 1.43 fold, respectively) compared to the diabetic group.

### 3.4. Effects of U, A, and Z Extracts and Their Combination (UAZ) on Serum Lipid Profile in Mice

The levels of plasma TC, TG, LDL-C, and HDL-C in normal and STZ mice are shown in Table 4, which were significantly increased in the diabetic group compared to the control group. These biochemical parameters were significantly decreased in the A, Z, U, and UAZ groups at 250 mg/kg dose each compared to the STZ groups. The effect was most significant in the UAZ group, which also had an increased HDL-C level compared to the diabetic control group.

### 3.5. Liver Toxicity Markers, ALP, AST, ALT, Lipid Peroxidation (LPO), and Lipid Hydroperoxides (LOOH) Levels in the Mice Serum

**Figure 3:**
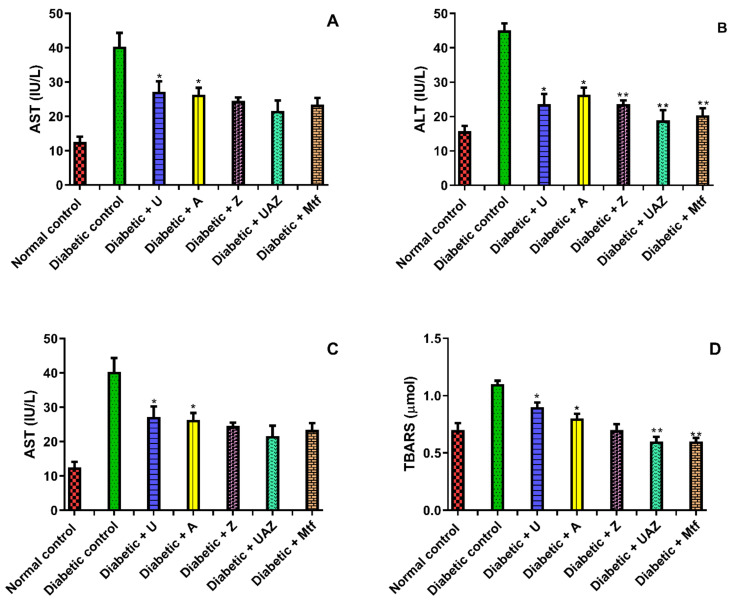
Effects of UZA, U, A, and Z treatment on marker in liver (**A**) ASTL; (**B**) ALT; (**C**) ALT; (**D**) TBARS levels; Data are presented as the means ± SD (n = 6). ** *p* < 0.001, * *p* < 0.05.

In the diabetic group, ALP, AST, ALT, and TBARS were significantly (*p* < 0.05) increased in comparison to the normal group. However, the activities of ALP, AST, TBRS, and ALT were significantly (*p* < 0.05) reduced in diabetic mice treated with U, A, and Z extracts. In contrast, UAZ treatment had a synergistic effect (Figure 3). The levels of plasma LPO and LOOH were significantly (*p* < 0.05) increased in STZ-treated mice compared to the normal animals. U, A, and Z extracts and the polyherbal formulation normalized the levels of LOOH in STZ-treated mice (Table 5).

### 3.6. Antioxidant Enzymes SOD, CAT, and Non-Enzymatic GSH in the Mice Liver

After the injection of STZ, there was a significant reduction in the content of antioxidant enzymes. There was a significant increase in the levels of SOD, CAT, and GSH in the mice liver when given 250 mg/kg of the extracts (Table 4).

### 3.7. Polyherbal Formulation Effect on the Viability, ROS, LPO, and Stimulated Insulin Secretion of Pancreatic β Cells Exposed to High Glucose

**Figure 4:**
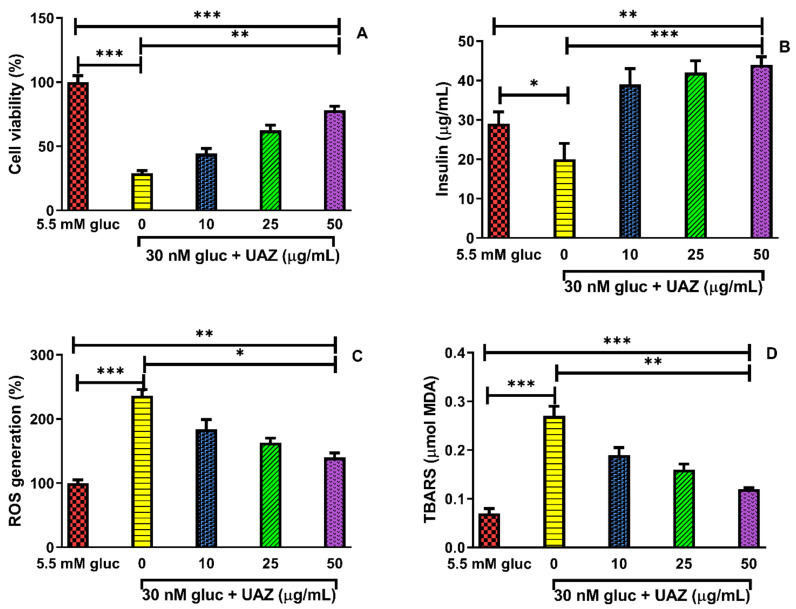
(**A**) UAZ enhance pancreatic b-cell viability when RINm5F cells were incubated in RPMI1640 medium containing glucose at 30 mM with or without various concentrations of (UAZ; 10, 25, and 50 µg/mL) for 48 h; (**B**) UAZ improved high glucose impaired insulin secretion; (**C**) Effect of UAZ on intracellular levels of reactive oxygen species (ROS) in high glucose-treated RINm5F cells; (**D**) TBARS generation in high glucose-treated INS-1 pancreaticβ-cells. The use of 5.5 mM glucose was representative of normal glucose conditions and the 30 mM glucose treatments represent high glucose conditions. Data are expressed as the mean ± SD, * *p* < 0.05, ** *p* < 0.01, and *** *p* < 0.001.

Pancreatic-β cells exposed to 30 mM glucose retained only 29% of viability, as compared to cells exposed to 5.5 mM glucose. However, the addition of UAZ (10–50 μg/mL) to RINm5F cells, resulted in a dose-dependent increase in viability of cells exposed to high glucose environment. Notably, the exposure to 50 μg/mL of UAZ along with high glucose treatment resulted in a significant increase in cell viability to 78.2%, Figure 4A.

We can observe two phenomena in this experiment: the addition of the extract to the cells in high glucose environment (1) counteracted the decrease of secreted insulin levels observed when the cells were exposed only to 30 mM glucose; (2) increased the secreted insulin levels, compared to 5.5 mM glucose, Figure 4B. To evaluate the antioxidant role of UAZ in pancreatic β cells, RINm5F cells were used to determine the level of ROS. As displayed in Figure 4C, the generation of intracellular ROS in RINm5F pancreatic β-cells increased significantly after treating with 30 mM glucose (236%) compared to those treated with 5.5 mM glucose. However, UAZ treatment reduced the levels of ROS in the cells cultured in high glucose in a dose-dependent manner, and 50μg/mL of UAZ produced a significant reduction in the intracellular ROS to 140%.

As shown in Figure 4D, the effect of UAZ on lipid peroxidation in RINm5F pancreatic β-cells cultured in high glucose was determined by measuring TBARS, a lipid peroxidation product. When RINm5F pancreatic β-cells were incubated for 48 h with 30 mM glucose, TBARS was significantly increased while 50 μg/mL of UAZ along with high glucose significantly inhibited TBARS formation, indicating its protective effect against lipid peroxidation.

### 3.8. Glucose Levels and Liver Enzymes in Zebrafish

As shown in Table 6, the group exposed to each extract and metformin has been shown to have low levels of the liver enzymes compared to the diabetic group; the extracts reduced the activity of the liver enzyme to the normal range. Adult zebrafish were treated with 4% glucose solution for four weeks. They showed glucose-induced basal hyperglycemia (187 mg/dl) after four weeks of administration (Table 6), and 50 mg/L of U, A, and Z extracts, UAZ and 25 mM of metformin treatment enhanced the onset of hyperglycemia and reduced basal glycemia to 57.2%, 60%, 62.6%, 66%, and 67%, respectively, making its levels equal to normal ones.

### 3.9. Hypolipidemic Effect in Zebrafish

In T2DM zebrafish groups, the levels of serum TC and TG were increased by 1.96 and 2.7 fold, respectively, compared to the normal group. After 4 weeks of administering 50 mg/L of U, A, and Z extracts, UAZ and 25 mM of metformin, the levels of serum TC and TG decreased by about 43% and 67.4% compared to the diabetic group.

## 4. Discussion

We found that the plant extracts analyzed showed anti-diabetic properties. This might be due to the presence of phenolic compounds which have been reported to have anti-diabetic effects. Examples include the following: ellagic acid that can reduce glucose and have anti-inflammatory, antioxidant, and anti-glycation effects. In addition, it prevents micro-and macrovascular diabetic complications [16]. Ferulic acid can markedly decrease blood glucose level, restore alterations in insulin signaling, ameliorate inflammatory cytokine release, and reduce protein tyrosine phospha tase1B (PTP1B) expression [17], *trans*-cinnamic acid that can improve pancreatic β-cell functionality, stimulate insulin secretion, and enhance reduction of glucose uptake of hepatic gluconeogenesis [18]. Gallic acid and *p*-Coumaric acid that ameliorate glucose tolerance, improve antioxidant status, increase the levels of PPARγ mRNA, adiponectin, decrease the level of TNF-α and lipid profile parameters [19]. Chlorogenic acid that possess hypoglycemic, hypolipidemic, anti-inflammatory, and antioxidant properties [20]. Syringic acid can revert both parameters of hyperinsulinemia and hyperglycemia [21].

Vanillic acid significantly decreases the levels of glucose, serum insulin, triglyceride, and free fatty acid [22].

In these extracts, flavonoids have also been found to have potential effects on diabetes such as kaempferol, which significantly improves blood glucose, insulin sensitivity, reduces hepatic glucose production, decreases pyruvate carboxylase, increases hexokinase activity, and decreases glucose-6 phosphatase activity in the liver [23]. Naringin reduces hyperglycemia, inhibits the proliferation of cells induced by high glucose, and decreases the expression of inflammatory that is mediated by NLRP3 through the NLRP3-caspase-1-IL-1β/IL-18 signaling pathway [24]. Rutin decreases blood glucose level, decreases levels of total cholesterol, low-density lipoprotein (LDL), HDL cholesterol, triglyceride, aspartate aminotransferase (AST), and alanine transaminase (ALT) [25] (Ganeshpurkar et al., 2017). Morin protects RINm5F cells from STZ-induced cell damage by triggering the phosphorylation of AMPK, thus resulting in the subsequent activation of FOXO_3_ and induction of catalase [26].

Treatment with the extracts significantly reversed the oxidative stress associated/induced changes, which could be due to an improvement in insulin secretion. The reduction of insulin secretion is the cause of high blood glucose levels, which is a characteristic of hyperglycemia. High blood glucose level and low secretion of insulin after administration of -are features of hyperglycemia, and the oral administration of the extracts for 4 weeks significantly reduced the fasting blood glucose level in the diabetic mice. This demonstrates the antidiabetic effect of these extracts. The glucose levels could have been reduced by increasing the utilization of glucose levels in muscles, potentiating the secretion of insulin, and reducing intestinal absorption [27].

The extracts administered increased the level of insulin after 4 weeks, which could be due to the normalization of pancreatic insulin or the insulin stimulatory activity of the phytochemical content of the extracts [28]. The excess glucose present in the plasma in diabetes mellitus tends to react with hemoglobin to produce HbA1c [29]. Data were comparable to metformin-treated mice. Chronic hyperglycemia is displayed in the glycation of Hb that leads to the formation of HbA1c. The increase in the levels of HbA1c in the experimental diabetic mice indicates the oxidation of sugars with extensive damage to both proteins and sugars in the circulation [30].

The significant increase in the level of hexokinase indicates stimulation of glycolysis in tissues with effective utilization of glucose removal from the circulation. G6PD plays an important role in β-cells’ survival and their functions. Hyperglycemia inhibits G6PD activity leading to gradual loss of β-cells [31]. The extracts administered increased the activity of G6PD in hepatic tissues, increasing glucose utilization via the pentose phosphate pathway.

Deficiency of insulin in hyperglycemic condition activates G6P and F1,6BP gluconeogenic enzymes. Glucose homeostasis is maintained by these enzymes; they ameliorate hepatic glucose utilization and hepatic glucose production in STZ-induced diabetic mice, which may be mediated by hepatic G6P activity [32]. Activation of 6BP and F1 catalyzes their dephosphorylation in the gluconeogenesis pathway and intervenes in gluconeogenic flux. The significant reduction in both G6P and F1 activities in the hepatic tissues of diabetic mice by the extract supplements led to the generation of endogenous glucose and modification of gluconeogenic flux. Phosphofructokinase is a glycolytic enzyme whose function is to regulate the rate of glucose utilization and is dysregulated as well as mutated in cancer. The level of G6P is determined by phosphofructokinase, which is connected to the G6P isomerase reaction operating at quasi-equilibrium. An increase in the phosphofructokinase activity can reduce G6P; therefore, hexokinase transforms are disinhibited, which consequently increases glucose utilization [33].

Hyperglycemia causes chronic damage and injury to different organs. Therefore, the achievement of good glycemic control is essential to prevent or delay diabetes complications. Herein, diabetic mice treated with UAZ showed a significant reduction in blood glucose levels increasing peripheral glucose utilization and decrease hepatic gluconeogenesis supported by increase the activity of hepatic glycolytic enzymes, such as hexokinase, phosphofructokinase, fructose-1, 6-bisphosphatase, glucose-6-phosphatase, and glucose-6-phosphatase dehydrogenase in the liver suggest that the hypoglycemic effect in part might be due to increased peripheral glucose utilization and decreased gluco-neogenesis in the liver through its insulin mimetic effect [33].

Insulin resistance influences glucose metabolism and lipid metabolism, leading to abnormal higher glucose and lipid contents. These cause hyperlipidemia and dyslipidemia, respectively, and are involved in metabolic disorders such as diabetes [34]. It has been shown the extracts can improve circulating lipid profiles in diabetic mice. UAZ, U, A, and Z extract supplements significantly enhanced blood lipid profile levels, showing that they have a hypolipidemic effect as they contain flavonoids.

The higher levels of ALP, AST, TBARS, and ALT in diabetic animals suggest a functional impairment of hepatic cell membranes and a cellular leakage which demonstrate the hepatotoxic effect of streptozotocin in diabetic mice [35]. The alterations observed in the hepatic enzymes ALP, AST, and ALT can be reversed using the extracts. This shows that they can protect the liver, particularly on membrane permeability due to their target oxidants, and consequently can function as free radical scavengers [36]. The extracts have the capacity to inhibit lipid peroxidation preventing STZ-induced oxidative stress and protecting β-cells. They can lead to increased secretion of insulin and decrease plasma glucose levels just as metformin [37]. Hyperglycemia increases LPO, contributing to the free radical-induced process. This leads to oxidative degradation of polyunsaturated fatty acids and elevated levels of LOOH in the plasma of diabetic mice. LPO is characteristic of chronic diabetes which is determined as TBARS found to be significantly inhibited through free radical scavenging effects.

High levels of lipid peroxidation are dangerous to the hepatic tissue and prejudice the capacity of antioxidants to eliminate excess ROS generation [38]. Antioxidant enzymes SOD, CAT, and the non-enzymatic GSH in the liver can reduce the production of free radicals by scavenging initiating radicals, chelating the transition metal catalysts, breaking chain reactions, and reducing concentrations of ROS [34]. The findings of this study demonstrate that the extracts led to a reduction in the levels of glutathione, superoxide dismutase, and catalase, and it is supported that liver deterioration is associated with oxidative stress [39]. U, A, and Z extracts and the polyherbal formulation increased the activity of antioxidant enzymes, which heightened the levels of non-enzymatic antioxidants (GSH) and both enzymatic (SOD, CAT) antioxidants while decreasing the activity of MDA in hepatic tissues.

Treating the RINm5F pancreaticβ-cells with high levels of glucose significantly reduced their viability. However, when treated with UAZ, a dose-dependent increase in cell viability was observed compared to the high glucose treatment, indicating that UAZ protects RINm5F cells from high glucose-induced cytotoxicity. Hyperglycemia deteriorates pancreatic insulin secretory capacity and peripheral insulin sensitivity [40]. UAZ was able to protect pancreatic β cells and improve insulin response. Increase in glucose levels stimulates insulin release, while exposure of pancreatic cells to high glucose conditions impairs both their insulin secretory function and cell viability [41]. Thus, due to these events, this study shows the ability of the extracts to ameliorate the viability and insulin secretion function of pancreatic β cells cultured with high glucose. High ROS levels generate oxidative stress, producing a variety of physiological and biochemical damages which impair metabolic function and lead to cell death [42]. The study findings indicate that exposure of RINm5F cells to glucose (30 mM) significantly increased intracellular ROS levels. Nevertheless, UAZ decreased high glucose-induced by ROS production.

Lipid peroxidation is known as a marker of cell damage through free radicals [43]. In this study, exposure to high glucose has been shown to induce lipid peroxidation in RINm5F cells, while UAZ effectively reduced TBARS formation. Plasma and mitochondrial membranes were mostly damaged by lipid peroxidation which produced low molecular weight end products, as MDA, formed through the oxidation of polyunsaturated fatty acids. It is demonstrated that UAZ can prevent TBARS formation associated with its antiperoxidative properties.

Results in T2DM zebrafish model confirm that UAZ could decrease their glycemia and liver enzyme levels after being exposed to glucose and also those found in STZ-nicotinamide diabetic mice. However, there was no mortality observed in the zebrafish group exposed to each extract and metformin, while in the diabetic group the mortality rate was 12%.

## 5. Conclusions

Medicinal plants are commonly used in the treatment of diabetes particularly due to their flavonoids and phenolic compounds. Multi-targeted polyherbal drug systems contain good combinations of phytochemicals that have synergistic effects in contrast to modern medicinal drugs. The study results show that UAZ or U, A, and Z given orally to diabetic mice displayed hypoglycemic and hypo-lipidemic effects by reducing liver weight and ameliorating the activities of hepatic enzymes such as fructose-1,6-bisphosphatase hexokinase, glucose-6-phosphatase, phosphofructokinase, alkaline phosphatase (ALP), alanine aminotransferase (ALT), and aspartate aminotransferase (AST) compared to the T2DM group. The extracts led to an increase in antioxidant enzymes such as superoxide dismutase (SOD), catalase (CAT), and a reduction in glutathione (GSH), oxidative stress biomarker including thiobarbituric reactive species (TBARS), and lipid hydroperoxides (LOOH) in the liver tissues of T2DM group over 4 weeks of treatment period compared to the STZ-induced diabetic group. They increased levels of insulin in serum and inhibited glycated hemoglobin (HbA1c). The extracts protect and perform antidiabetic activities in pancreatic β-cell culture (RINm5F cells). They lower lipid, reduce liver enzymes and hypoglycemic activities in diabetic zebrafish. They probably have antidiabetic effects by interacting with hepatic glycolytic and gluconeogenic enzymes and through their hypolipidemic, antioxidant, and insulinotropic properties. It is also shown that the polyherbal formulation has high anti-diabetic properties due to its high compound contents and synergistic effect. It is a potential therapeutic agent that could have an important role in the management of metabolic disorders such as diabetes.

## Figures and Tables

**Table 1:** Identification and characterization of the flavonoids and phenolic acids from *U. dioica, Z. officinale*, and *A. graveolens*.

*U. dioica*			
Total flavonoids RE/100 g 0.26 ± 0.007			
Characterization			
Compound	RT min	λmax (nm)	[M+H]^+^ *m*/*z*
Ellagic acid	39.9	254, 368	302, 257, 249, 125
Ferulic acid	33.5	235,322	193, 177, 148, 133
Isorhamnetin-3-*O*-rutinoside	23.4	250, 268, 342	623, 471, 363, 447, 315, 271, 227, 150
Kaempferol-3-*O*-glucoside (astragalin)	25.3	265, 346	447, 285, 256, 211, 151, 117
Myricetin	26.2	208, 253, 368	369, 319, 193, 147, 133
Naringin	28.5	227, 283	579, 459, 339, 271, 235, 151, 134
Quercetin-3-*O*-glucoside	24.9	256, 344	465, 303, 300, 214, 154
Rutin	32.1	254, 354	610, 509, 343, 302, 300, 271, 151
*Z. officinale*			
Total flavonoids RE/100 g 4.59 ± 0.97			
Characterization			
Ferulic acid, naringenin, rutin			
*trans*-cinnamic acid	22.6	203, 273	148, 141, 131, 03, 102, 91, 77
Epicatechin	17.54	220, 280	291, 245, 212, 87, 151, 123, 109
Fisetin	15.3	360	286, 285, 257, 163, 135
Gallic acid	9.6	209, 266	170, 169, 153, 125
Kaempferol	26.7	265, 365	287, 258, 213, 185, 183, 137, 121
Morin	8.7	260, 360	303, 285, 229, 213, 177, 121
Quercetin	42	258, 380	303, 286, 257, 229, 201, 165, 153, 137
Vanillic acid	17.1	259, 252	312, 297, 282, 223, 193, 165, 126
*A. graveolens*			
Total flavonoids RE/100 g 0.824 ± 0.039			
Characterization			
Compound	RT min	λmax (nm)	[M+H]^+^ *m*/*z*
Ferulic acid, gallic acid, quercetin, rutin, vanillic acid			
Caffeic acid	18.4	210, 240, 325	181, 163, 145, 135, 107
Chlorogenic acid	14.6	325, 294, 216	353, 221,191, 161, 178, 134
*p*-Coumaric acid	23.7	212, 283	165, 147, 133, 119, 91
Luteolin	21	264, 356	285, 175, 167, 133
Syringic acid	19.2	275	198, 183, 127

The total flavonoids of each of the three extracts (U, Z and A) are shown in Table 1. *Z. officinale* has the highest level of flavonoids content (4.59 RE/100 g), followed by *A. graveolens* (0.824 RE/100 g), and *U. dioica* (0.26 RE/100 g), while UAZ has the highest level of total flavonoids (5.78 RE/100 g) compared to the other extracts.

**Table 2:** Fasting blood sugar in normal and streptozotocin-induced diabetic mice with polyherbal formulation (UAZ), and extracts from *U. dioica* (U), *A. graveolens* (A), and *Z. officinale* (Z) in days 0, 2 weeks, and 4 weeks of treatment.

Group	First Day	Week 2	Week 4
Control	90 ± 4.13	91 ± 3.78	89 ± 5.12
Diabetic Control	279 ± 6.74	310 ± 9.47	350 ± 9.38
Diabetic + U	285 ± 8.25	225 ± 5.38 ^a,b^	161.7 ± 3.41 ^a,b^
Diabetic + A	275 ± 6.61	215 ± 7.09 ^a,b^	155.4 ± 2.52 ^a,b^
Diabetic + Z	278 ± 7.40	200 ± 6.21 ^a,b^	145.6 ± 1.97 ^a,b^
Diabetic + UAZ	271 ± 5.33	191 ± 4.88 ^b^	112 ± 4.88 ^b^
Diabetic + Mtf	280 ± 4.98	186 ± 5.64 ^b^	90 ± 5.64 ^b^

Values are showed as mean ± SD (n = 8). Significant difference compared with the control group ^a^ *p* < 0.01 and compared vs. diabetic control group ^b^ *p* < 0.05.

**Table 3:** Effect of U, A, and Z and UAZ on activities of hexokinase, phosphofructokinase, fructose-1, 6-bisphosphatase, glucose-6-phosphatase, and glucose-6-phosphatase dehydrogenase.

Groups	Hexokinase (U*/g Protein)	Phosphofructokinase (mMol/min/mg Protein)	Fructose-1, 6-Bisphosphatase (U**/g Protein)	Glucose-6-Phosphatase (U*/g Protein)	Glucose-6-Phosphatase Dehydrogenase (X10^−4^ mIU/mg Protein)
Normal control	107.16 ± 3.28 ^a^	29.43 ± 3.67 ^a^	0.386 ± 0.07 ^a^	0.136 ± 0.04 ^a^	3.710 ± 0.13 ^a^
Diabetic control	67.23 ± 5.41 ^b^	20.97 ± 2.12 ^b^	0.642 ± 0.08 ^b^	0.281 ± 0.03 ^b^	2.689 ± 0.22 ^b^
Diabetic + U	80.40 ± 4.98 ^c^	26.36 ± 1.98 ^c^	0.538 ± 0.09 ^d^	0.182 ± 0.04 ^d^	2.903 ± 0.45 ^c^
Diabetic + A	89.35 ± 3.95 ^c^	27.10 ± 4.19 ^c^	0.476 ± 0.05 ^c^	0.175 ± 0.04 ^c^	2.981 ± 0.32 ^c^
Diabetic + Z	91.65 ± 4.56 ^d^	28.17 ± 1.24 ^c^	0.435 ± 0.06 ^c^	0.168 ± 0.04 ^c^	3.124 ± 0.15 ^a^
Diabetic + UAZ	101.87 ± 6.31 ^a^	32.25 ± 3.28 ^a^	0.397 ± 0.05 ^a^	0.151 ± 0.06 ^a^	3.372 ± 0.38 ^a^
Diabetic + Mtf	98.54 ± 4.87 ^d^	30.12 ± 3.28 ^a^	0.400 ± 0.02 ^a^	0.159 ± 0.07 ^a^	3.418 ± 0.45 ^a^

Data are expressed as mean ± SD (n = 6). Values with a common superscript letter differ significantly at *p* < 0.05; U* moles of glucose phosphorylated per minute; U** moles of inorganic phosphate liberated per hour.

**Table 4:** Effects of U, A, and Z and UAZ on serum contents of TG, TC, LDL-C, and HDL-C for lipid profile in mice.

Groups	TG (mmol/L)	TC (mmol/L)	LDL-C (mmol/L)	HDL-C (mmol/L)
Normal control	1.8 ± 0.4	1.7 ± 0.3	0.47 ± 0.13	0.78 ± 0.07
Diabetic control	3.4 ± 0.3 ^a^	3.3 ± 0. 4 ^a^	1.67 ± 0.12 ^a^	0.69 ± 0.05 ^a^
Diabetic + U	2.6 ± 0.1 ^d^	2.8 ± 0.2 ^b^	0.98 ± 0.10 ^d^	0.72 ± 0.01 ^b^
Diabetic + A	2.4 ± 0.3 ^b^	2.5 ± 0.3 ^b^	0.87 ± 0.09 ^b^	0.74 ± 0.06 ^b^
Diabetic + Z	2.1 ± 0.2 ^b^	2.0 ± 0.2 ^c^	0.72 ± 0.13 ^b^	0.76 ± 0.03 ^c^
Diabetic + UAZ	1.7 ± 0.4 ^c^	1.7 ± 0.3 ^d^	0.48± 0.11 ^c^	0.78± 0.01 ^c^
Diabetic + Mtf	1.8 ± 0.2 ^c^	1.8 ± 0.2 ^d^	0.49± 0.10 ^c^	0.79± 0.04 ^c^

Values are expressed as means ± SD (n = 6); Values with a common superscript letter differ significantly at *p* < 0.05.

**Table 5:** Effect of U, A, and Z and UAZ on lipid hydroperoxides (LOOH) in serum and hepatic contents in antioxidant enzymes SOD, CAT, and antioxidant molecules GSH in ZTZ-induce diabetic mice.

Groups	Serum Liver			
	LOOH (1 × 10^−5^ mmol/dL)	SOD (U/mg Protein)	CAT (U/mg Protein)	GSH (mg/g Protein)
Normal control	10.4 ± 0.9	285.1 ± 8.96	90.1 ± 6.41	10.9 ± 1.34
Diabetic control	15.9 ± 1.1 ^a^	157.2 ± 7.66 ^a^	32.4 ± 2.85 ^a^	4.8 ± 0.87 ^a^
Diabetic + U	12.2 ± 1.8 ^c^	180.4 ± 9.32 ^b^	64.8 ± 5.12 ^b^	5.64 ± 1.56 ^c^
Diabetic + A	11.36 ± 2.3 ^d^	201.6 ± 6.85 ^d^	70.1 ± 3.56 ^c^	6.12 ± 0.87 ^d^
Diabetic + Z	10.52 ± 1.6 ^d^	220.3 ± 8.63 ^d^	74.5 ± 4.89 ^d^	6.48 ± 0.99 ^d^
Diabetic + UAZ	9.76 ± 0.7 ^c^	281.3 ± 10.0 ^c^	87.1 ± 4.61 ^e^	8.12 ± 1.10 ^e^
Diabetic + Mtf	9.78 ± 1.5 ^c^	279.4 ± 9.21 ^c^	85.2 ± 2.16 ^e^	6.99 ± 0.87 ^d^

Values are expressed as means ± SD (n = 6); Values with a common superscript letter differ significantly at *p* < 0.05.

**Table 6:** Effect of U, A, and Z, and UZA on glucose in serum and liver enzymes in glucose-induced diabetic zebrafish.

Groups	Serum			Liver		
	Glucose mg/dL	TG (mmol/L)	TC (mmol/L)	ALP (IU/L)	ALT (IU/L)	AST (IU/L)
Normal control	60 ± 2.3	1.6 ± 0.01	2.6 ± 0.04	0.05 ± 0.003	115 ± 5.36	36.2 ± 2.04
Diabetic control	187 ± 5.4 ^a^	4.3 ± 0.03 ^a^	5.1 ± 0.08 ^a^	0.34 ± 0.004 ^a^	243 ± 7.21 ^a^	49.6 ± 3.42 ^a^
Diabetic + U	80 ± 3.6 ^c^	2.0 ± 0.02 ^c^	4.0 ± 0.06 ^b^	0.16 ± 0.001 ^b^	205 ± 6.48 ^c^	40.5 ± 1.56 ^b^
Diabetic + A	75 ± 5.5 ^d^	1.9 ± 0.04 ^b^	3.5 ± 0.07 ^c^	0.13 ± 0.003 ^c^	200 ± 7.36 ^d^	36.1 ± 2.50 ^c^
Diabetic + Z	70 ± 2.8 ^d^	1.7 ± 0.03 ^b^	3.2 ± 0.02 ^c^	0.10 ± 0.005 ^d^	196 ± 4.94 ^b^	34.7 ± 1.83 ^c^
Diabetic + UAZ	64 ± 4.3 ^b^	1.4 ± 0.02 ^d^	2.9 ± 0.01 ^d^	0.07 ± 0.002 ^e^	185 ± 4.76 ^e^	30.5 ± 2.57 ^d^
Diabetic + Mtf	62 ± 3.6 ^b^	1.4 ± 0.0.4 ^d^	3.0 ± 0.04 ^c^	0.06 ± 0.003 ^e^	199 ± 5.28 ^b^	28.5 ± 1.90 ^e^

Values are expressed as means ± SD (n = 6); Values with a common superscript letter differ significantly at *p* < 0.05.

## Data Availability

Data available in a publicly accessible repository.
